# Retraction: Caffeine Positively Modulates Ferritin Heavy Chain Expression in H460 Cells: Effects on Cell Proliferation

**DOI:** 10.1371/journal.pone.0346201

**Published:** 2026-04-02

**Authors:** The PLOS One Editors

After publication of this article [1], concerns were raised regarding Figures 1, 3, 4, and 6. Specifically, the following panels appear similar despite representing different experimental conditions:

- Fig 1C γ-TUB lanes 2-3, Fig 3A γ-TUB resized horizontally and vertically, and Fig 4A γ-TUB lanes 1-2.
- Fig 1C γ-TUB lanes 2-4 and Fig 4A γ-TUB lanes 1-3.
- Fig 3E CCND1 lane 1 and Fig 3E γ-TUB lane 1 flipped horizontally.
- Fig 6B Y-TUBULIN and Fig 6D Y-TUB lanes 3-4 flipped vertically.

Co-first author FB stated that the γ-tubulin panel in Fig 4A is from the same experimental series as Fig 1C where H460 cells were treated with 20, 40, 80, and 120 µM caffeine, and this was used to evaluate FHC (Fig 4A), p53 and CCND1 (Fig 1C). They stated that only the 20, 40, and 80 µM caffeine conditions were reported in [1], and that a figure preparation error occurred in Fig 4A where the 120 µM condition was inadvertently included. The *PLOS One* Editors therefore consider the concerns with similarities between the Fig 1C and Fig 4A γ-TUB panels resolved.

During post-publication discussions several original western blot images were provided for the remainder of the above concerns, however some of these do not appear to match the corresponding panels in [1]. As such, PLOS considers the concerns for Fig 3A γ-TUB, Figs 3E γ-TUB and CCND1, and Figs 6B and 6D γ-TUB unresolved.

In light of the above concerns which call into question the reliability of the results in [1], the *PLOS One* Editors retract this article.

FB, AMB, MCF, and FC did not agree with the retraction. FZ and GC either did not respond directly or could not be reached.
